# [Retracted] Knockdown of circ_0001883 may inhibit epithelial‑mesenchymal transition in laryngeal squamous cell carcinoma via the miR‑125‑5p/PI3K/AKT axis

**DOI:** 10.3892/etm.2024.12417

**Published:** 2024-02-05

**Authors:** Fu Chen, Zheng Lao, Haiyan Zhang, Jie Wang, Shengzi Wang

Exp Ther Med 22:1007, 2021; 10.3892/etm.2021.10440

Following the publication of this paper, it was drawn to the Editors’ attention by a concerned reader that the tumour images shown in Fig. 6A, Transwell invasion assay data shown in Fig. 5E and certain of the MMP3 data shown in Fig. 4G were strikingly similar to data appearing in different form in other articles written by different authors at different research institutes that had either already been published elsewhere prior to the submission of this paper to *Experimental and Therapeutic Medicine*, or were under consideration for publication at around the same time.

In view of the fact that certain of these data had already apparently been published prior to the submission of this article for publication, the Editor of *Experimental and Therapeutic Medicine* has decided that this paper should be retracted from the Journal. The authors were asked for an explanation to account for these concerns, but the Editorial Office did not receive a reply. The Editor apologizes to the readership for any inconvenience caused.

